# Occupational Burnout levels in Emergency Medicine–a stage 2 nationwide study and analysis

**Published:** 2010-11-25

**Authors:** F Popa, R Arafat, VL Purcărea, A Lală, O Popa–Velea, G Bobirnac

**Affiliations:** * ‘Carol Davila’ University of Medicine and Pharmacy,Bucharest Romania; ** Romanian Ministry of Health, Bucharest Romania

**Keywords:** Emergency Medicine, burnout syndrome, depression, coping mechanisms, work environment, human resources, stress, psychological traits

## Abstract

**Introduction:** The first stage of this nationwide study and analysis of the occupational burnout and psychological risk parameters showed a high consistency of emotional exhaustion, depersonalization and low personal accomplishment for doctors working in Emergency Departments and Emergency and Resuscitation Services. These workers were then set in the highest risk group for burnout syndrome and depression. This stageⅡ of our research will focus on those two groups analyzing causal factors, coping mechanisms and possible repercussions of these findings.

**Material and methods:** Demographics: We have issued a total of 272 surveys from which we have received a total of 263 complete and valid ones (n=263, response rate=96, 69%).

**Instruments:** The Maslach Burnout Inventory–Human Services Survey MBI–HSS is an instrument designed to assess the three components of the burnout syndrome: emotional exhaustion (EE), depersonalization (DP), and reduced personal accomplishment (PA). The **COPE** questionnaire is a 52 item addressing different ways of coping with stress. The Center for Epidemiologic Studies Depression Scale **(CES-D)** has been shown to be a reliable measure in assessing the number, types, and duration of depressive symptoms across racial, gender, and age categories.

**Results and discussion**: Results were not correlated with gender, age or marital status, but an important correlation was found with professional experience in the Emergency Departments. We have shown that during the first 4 years of experience, the EE factor has been at a satisfying average of 2.4, this variable rising to an average of 2.85 after another 3 years of work. The same type of correlation was found with the CES–D results.

**Conclusions:** Of the two surveyed groups, the EMD group showed higher values for all risk parameters and low personal accomplishment on the MBI–HSS survey. Also, emotional exhaustion and depression were found to have a powerful correlation with work experience. Coping mechanisms were found to be invariable to the general population, with a slight incline towards active coping and behavioral disengagement

## Introduction

The first stage of this nationwide study and analysis of the occupational burnout and psychological risk parameters showed a high consistency of emotional exhaustion, depersonalization and low personal accomplishment for doctors working in Emergency Departments and Emergency and Resuscitation Services [1]. These workers were then set in the highest risk group for burnout syndrome and depression. This stage Ⅱ of our research will focus on those two groups analyzing causal factors, coping mechanisms and possible repercussions of these findings.

In the background, most studies linking the specialist's increased professional stress levels with a low quality medical act have a specific reference to emergency medicine. The average percentage given for doctors that have a higher than normal stress level has been an over–time constant in the area of 28%, in comparison with the 18% shown by the general population[2]. What has shown a clear change in the last years is the openness of the doctors to admit the existence of these problems and discuss them with colleagues or take part in group sessions of therapy. [3]

The far most important factor has been established as the lack of sleep on behalf of the doctor. This aspect has recently been given a high level of attention both by researchers and by management branches of the medical world. In the US and other countries following US protocols the maximum on–call period for an EM specialist has been dropped to 12 hours, half of that established for all other specialties, in recognition of the increased level of both physical and psychical stress that the doctor is subjected to. This being said, there is still no measurement taken that prohibits the medic to do overtime in private clinics or other hospitals, therefore a lot of controversies have arisen about the exact efficiency of these measurements.[5,4]

Either individual or conjectural, professional stress sources for EM specialists arise from many aspects of their activities. Among the most discussed are self criticizing attitudes, lack of communication, deficiencies in team–work and lack of professional support, deficient financial rewards, lack of personal time and the lack of self esteem caused by a faulty and unfounded hierarchy that places emergency medical specialists at the bottom of the list.

## Material and method

### Demographics

We have issued a total of 272 surveys from which we have received a total of 263 complete and valid ones (n=263, response rate=96, 69%). All surveys were anonymous; subjects were randomly selected from the Emergency Departments of state hospitals and were instructed to complete them individually, in their free time. Only M.D. degree subjects were chosen either with or without specialization in Emergency Medicine or Critical Care.

Demographics included age, gender, marital status, children, professional status and work experience. The survey contained detailed instructions on how to complete the demographics and questionnaires, and also offered the subjects the option to receive their own results via e–mail written on a part of the survey that would be detached after the analysis for anonymity purposes.

### Instruments

The Maslach Burnout Inventory–Human Services Survey MBI–HSS is an instrument designed to assess the three components of the burnout syndrome: emotional exhaustion (EE), depersonalization (DP), and reduced personal accomplishment (PA). There are 22 items, which are divided into three subscales. The items are written in the form of statements about personal feelings or attitudes (e.g., ‘I feel burned out from my work’, ‘I don't really care what happens to some recipients’). The items are answered in terms of the frequency with which the respondent experiences these feelings, on a 7–point, fully anchored scale (ranging from 0, ‘never’ to 6, ‘every day’). [7]

The reliability coefficients for the **MBI–HSS** were based on samples that were not used in the item selections to avoid any improper inflation of the reliability estimates. Internal consistency was estimated by Cronbach's coefficient alpha (n=1,316). The reliability coefficients for the subscales were the following: 0.90 for Emotional Exhaustion, 0.79 for Depersonalization, and 0.71 for Personal Accomplishment. The standard error of measurement for each subscale is as follows: 3.80 for Emotional Exhaustion, 3.16 for Depersonalization, and 3.73 for Personal Accomplishment. [8,9]

The **COPE** questionnaire is a 52 item addressing different ways of coping with stress. Items are rated on a 4–point scale ranging from 1 (I usually don't do this at all) to 4 (I usually do this a lot). These items are then analyzed as 13 different subscales, from which, for the efficacy of the study, we have selected 6:  active coping, denial, seeking social support for emotional reasons, behavioral disengagement, substance abuse disengagement and seeking social support for instrumental reasons.[10]  

The Center for Epidemiologic Studies Depression Scale **(CES-D)** has been shown to be a reliable measure for assessing the number, types, and duration of depressive symptoms across racial, gender, and age categories. [11]. Scores may vary from 0 to 80, with a risk threshold at 50, and risk score values being over 60. High internal consistency has been reported with Cronbach's alpha coefficients ranging from 0.85 to 0.90 across studies. Concurrent validity by clinical and self–report criteria, as well as substantial evidence of construct validity has been demonstrated [13]. 

Final analysis of surveys was made by personnel specialized in psychometric sciences, statistics were made by using Microsoft Excel software and SPSS software, only percentages and linear regression methods were used, all results verify a 95% confidence interval. 

## Results and discussion

**Graph 1:**
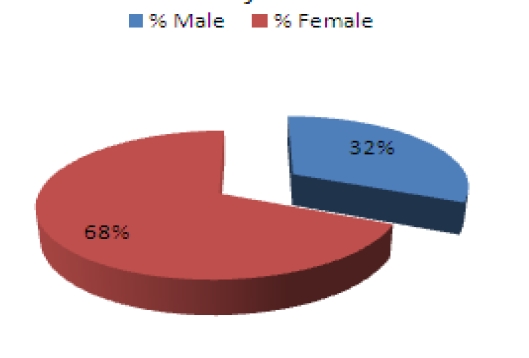
Gender percentages among subjects

**Graph 2:**
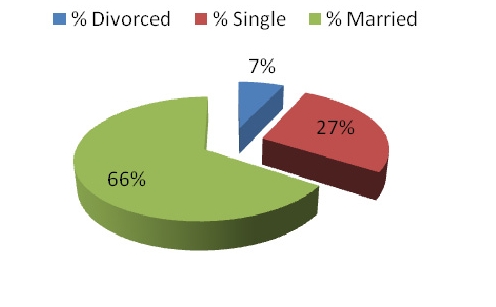
Marital Status among Subjects

**Graph 3:**
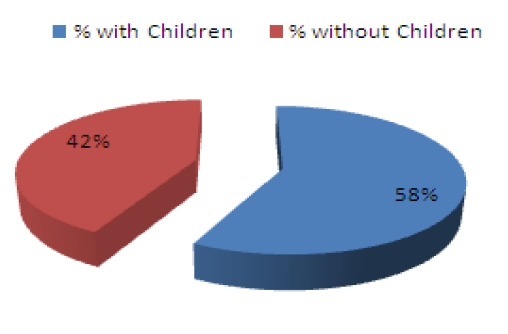
Percetange of subject with/without children

**Graph 4:**
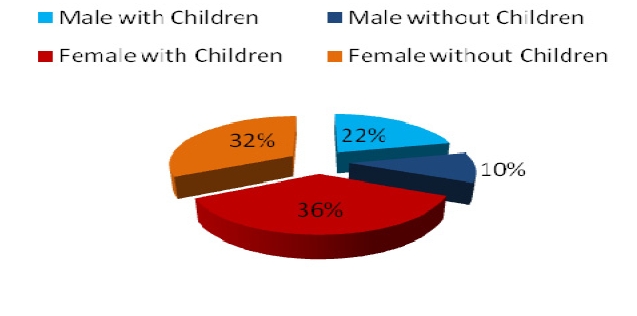
Child siblings

Average age among subjects was 39 years old with an average work experience of 7,4 years. Gender distribution was 68% male and 32% female (Graph 1). Marital status (Graph 2) shows a higher percentage of married subjects (66%) than the single ones (27%), while divorced (7%) rates are lower than the general population shown to be close to 11% [6]. Only 42% of the subjects have children (Graph 3), and further results show that there is a higher percentage of male workers who have children, which may be due to the fact that an important percentage of females change specialties or leave the Emergency Department after having a child (Graph 4).

Professional categories include Emergency Department physicians (EMD), SMURD service doctors (SMU). Also, we have added a checkbox for those who have a private practice job besides the regular hospital working hours (Table 1). 

**Table 1:** Number of subjects by professional category and private practice variable

nEMD	186
nSMU	87
nPRV	28

Results state that 70,07% of subjects worked only in the Emergency Department, while 29.03% had also mobile unit (SMURD) competences. Moreover, an important fact to be noted is that 10.64% of the surveyed workers also have a private practice job besides the state hospital job, adding to a total of 12 to 16 working hours per day.

Average MBI–HSS results confirm those in the first stage of our study [1], showing a higher rate of emotional exhaustion (EE) and depersonalization (DP) among EMD and SMU subjects than all other studied groups of emergency medical personnel. The EE factor was higher in EMD subjects (2.79) than in the SMU (2.16) reversely correlated with the professional accomplishment (PA) factor (Table 2). Also, the DP factor is higher in the EMD group, making these subjects the most vulnerable group for professional stress and burnout syndrome. 

**Table 2:** MBI–HSS subscales average results stratified by professional category

	GEN	EMD	SMU
EE	2,594	2,794	2,160
PA	4,062	4,075	4,034
DP	1,528	1,608	1,353

Results were not correlated with gender, age or marital status, but an important correlation was found with professional experience in the Emergency Departments (Table 3). We have shown that during the first 4 years of experience, the EE factor has been at a satisfying average of 2.4, this variable rising to an average of 2.85 after another 3 years of work. Also, the percentage of personnel showing higher values of EE have been shown to rise in the same parameters (Table 6) from an average of 10,89% showing EE>4 before the 4^th^ year of work experience and to 17,36% before the 7^th^. Work experience has a powerful influence on the burnout liability of emergency department doctors. Out of all work environment factors, experience time and patient flow were found to be the most important in setting the risk behavior pattern on these subjects[12].

The same correlation was found with the CES-D results. Average CES–D results do not vary significantly with age, gender or marital status, but work experience in the emergency department, again, shows an increase in both parameter average values (Table 5) and percentage of personnel showing risk values (Table 6). Burnout and depression have been shown to lower the quality of life, work environment satisfaction [13] and even quality of medical care [14]. 

Coping strategies as surveyed with the short version of the COPE questionnaire do not show a powerful alignment to certain coping mechanisms, although from the 6 selected (active coping, denial, seeking social support for emotional reasons, behavioral disengagement, substance abuse disengagement and seeking social support for instrumental reasons), the highest prevalence seems to be for active coping and behavioral disengagement (Table 7). Substance abuse is in the same limits as all other studied coping mechanisms, which confirms medical workers’ tendency to use alcohol and nicotine for disengagement purposes[15, 16].

**Table 3:** Variation of EE value averages correlated with professional experience in the EMD

EE value	Experience < 4 years	Experience > 4 years	Experience < 7 years	Experience > 7 years
Average	2,4	2,67	2,36	2,85

**Table 4:** Variation of EE factor value percentages correlated with work experience in the Emergency Department

EE value	Experience < 4 years	Experience > 4 years	Experience < 7 years	Experience > 7 years
>2	51,49%	70,11%	55,56%	75,21%
>3	27,72%	37,35%	25,19%	46,28%
>4	10,89%	13,21%	8,15%	17,36%

**Table 5:** Average CES–D results among subjects stratified by work experience

Experience	Experience < 4 years	Experience > 4 years	Experience < 7 years	Experience > 7 years
CES–D Average	18,10	18,95	17,30	20,23

**Table 6:** High risk CES–D results among subjects stratified by work experience

CES–D result	Experience < 4 years	Experience > 4 years	Experience < 7 years	Experience > 7 years
> 40	0,072	0,0574	0,051	0,074
> 50	0,036	0,0056	0,022	0,008

**Table 7:** COPE results stratified by coping mechanism

Coping Mechanism	Gen avg	EMD avg	SMU avg
Active coping	2,386	2,394	2,367
Denial	2,197	2,118	2,370
Seeking social support for emotional reasons	2,119	2,112	2,133
Behavioral disengagement	2,417	2,432	2,386
Substance abuse	2,224	2,244	2,179

## Conclusions

Of the two surveyed groups, the EMD group showed higher values for all risk parameters and low personal accomplishment on the MBI–HSS survey. Also, emotional exhaustion and depression were found to have a powerful correlation with work experience. Possible explanations for these findings might be linked to different organizations of work within the studied groups (e.g. aspects such as patient flow, crowding, working hours) but also to different individual characteristics, such as social development. None of the studied factors were found to be related with gender, age or marital status.

Coping mechanisms were found to be invariable to the general population, with a slight incline towards active coping and behavioral disengagement.

Study limitations include the risk of conformism for some subjects (due to the vertical top–down distribution of questionnaires), a relatively low n–value in comparison to the first stage of our study due to the scarcity of subjects and their geographical distribution, but the powerful correlations between the two separate studies that form the stages of our research may compensate for these limitations.
